# Acute onset bilateral hearing loss in dorsomedial pontine hemorrhage: A Case report

**DOI:** 10.1097/MD.0000000000016902

**Published:** 2019-08-23

**Authors:** Masashi Hoshino, Hisanao Akiyama, Satoru Kashima, Kaima Soga, Takahiro Shimizu, Yasuhiro Hasegawa

**Affiliations:** Department of Internal Medicine, Division of Neurology, St. Marianna University School of Medicine, Kawasaki, Kanagawa, Japan.

**Keywords:** acute onset hearing loss, auditory neuropathy, pontine hemorrhage

## Abstract

**Rationale:**

Nontraumatic pontine hemorrhage represents approximately 10% of all cases of nontraumatic intracranial hemorrhage. The predominant cause and symptom of pontine hemorrhage are hypertension and disturbance of consciousness, respectively.

**Patient concerns:**

A 64-year-old man was transported to hospital by ambulance for sudden articulation disorder and right leg paralysis. He was neurologically alert with mild dysarthria, right leg paralysis, and increased deep tendon reflex in all limbs.

**Diagnosis:**

Plain head computed tomography showed an approximately 1.5 mL hemorrhage in the dorsomedial pons.

**Intervention:**

He was admitted to the Stroke Care Unit and received antihypertensive therapy.

**Outcomes:**

Around the time of admission, bilateral hearing loss suddenly developed with poorly defined wave V shown bilaterally on auditory brainstem response testing. As the hematoma subsequently resolved, the bilateral hearing loss recovered sufficiently to enable everyday conversation.

**Lessons:**

We report herein a rare case of acute onset bilateral hearing loss caused by nontraumatic pontine hemorrhage. Pontine hemorrhage is often associated with disturbance of consciousness; however, care is required as latent communication disorder due to impaired hearing is possible regardless of the state of consciousness.

## 1 Introduction

Nontraumatic pontine hemorrhage represents approximately 10% of all cases of nontraumatic intracranial hemorrhage.^[1]^ The predominant cause and symptom of pontine hemorrhage are hypertension and disturbance of consciousness, respectively. As there are fundamentally no surgical options, treatment is usually conservative and outcomes are poor with a mortality rate of 40% to 60%.^[2]^ We report herein a rare case of dorsomedial pontine hemorrhage without change in consciousness in which the patient presented acute onset bilateral hearing loss immediately after hemorrhage onset that recovered as the hematoma subsequently resolved.

## 2 Case report

A 64-year-old man suddenly experienced articulation disorder and right leg paralysis immediately after exercise and was transported to our hospital by ambulance. He had a history of hypertension and was receiving oral combination antiplatelet therapy (100 mg aspirin and 75 mg clopidogrel) due to recent coronary stenting for angina.

On arrival, his blood pressure was 223/103 mm Hg. Neurologically, he was alert (Glasgow Coma Scale, E4V5M6) without ocular deviation or anisocoria. He presented mild dysarthria, right leg paralysis corresponding to Manual Muscle Test 4, and increased deep tendon reflex in all limbs (without laterality). No pathological reflexes were elicited and no sensory or coordination abnormalities were observed. His National Institutes of Health Stroke Scale score was 1.

**Figure 1:**
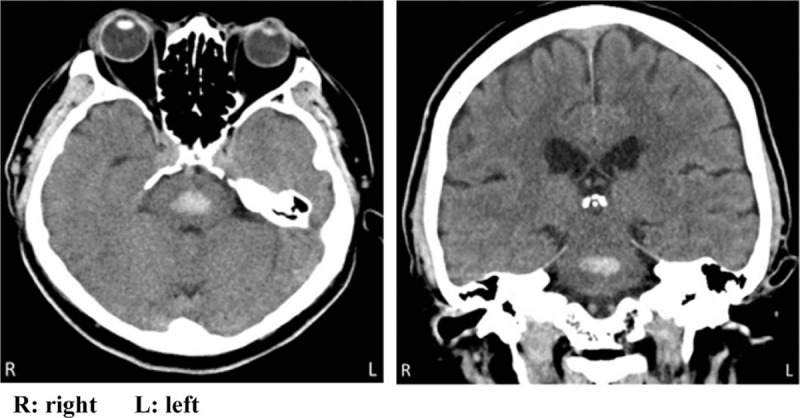
The axial and coronal images of the initial brain computed tomography showed an acute phase hemorrhage (volume 1.5 mL) of the central pons.

**Figure 2:**
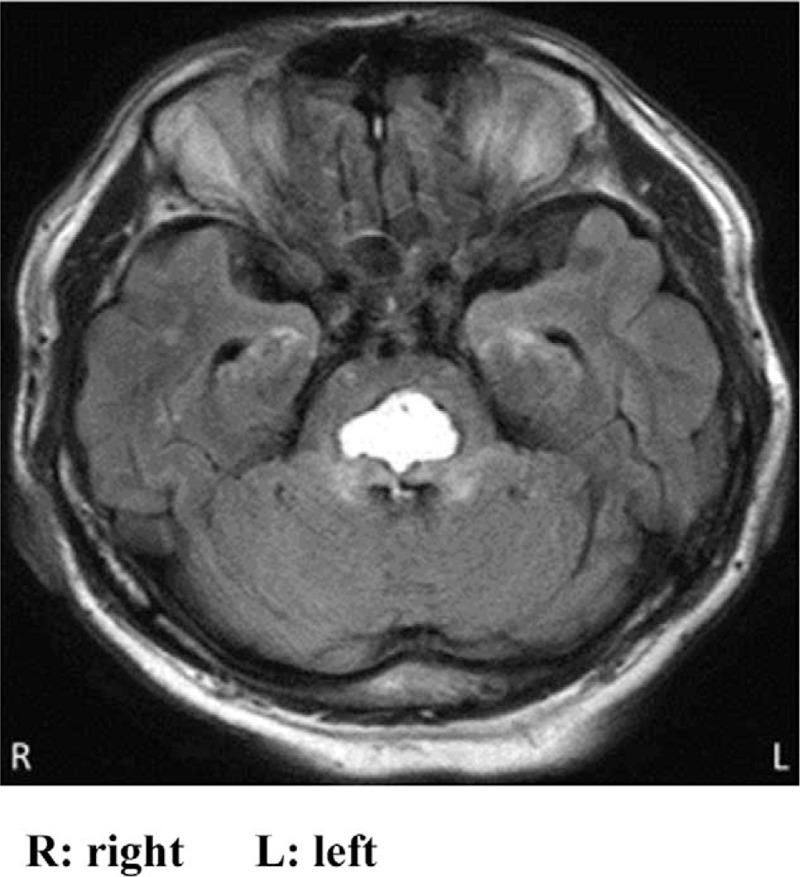
The axial image of the initial brain magnetic resonance imaging (T2 FLAIR) showed pontine hemorrhage with peripheral edema. FLAIR = fluid-attenuated inversion recovery.

Plain head computed tomography (CT) performed immediately after arrival showed an approximately 1.5 mL oval hemorrhage in the dorsomedial pons (Fig. 1). Thin slice imaging of the brainstem using plain head magnetic resonance imaging revealed hematoma in the dorsal lower pons and surrounding edematous changes (Fig. 2). On laboratory testing, platelet counts and coagulation profile were normal while serum anti-neutrophil cytoplasmic antibodies were negative. Head magnetic resonance angiography demonstrated no aneurysm in the major intracranial arteries; however, microbleeds were observed in the bilateral basal ganglia on fast field echo and hypertensive pontine hemorrhage was diagnosed.

**Figure 3:**
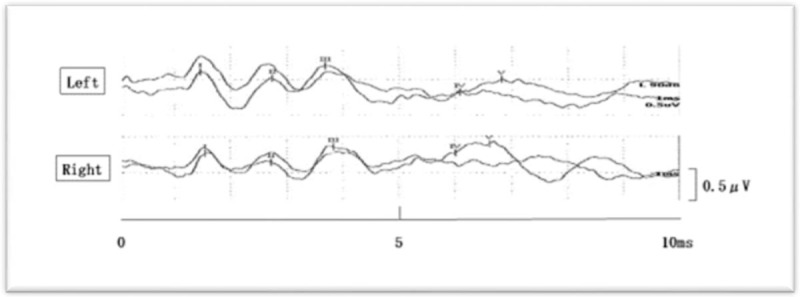
Auditory brainstem responses show poorly defined V waveforms.

Antihypertensive therapy was immediately initiated and he was admitted to the Stroke Care Unit; however, around this time, he suddenly developed bilateral hearing loss and left tinnitus. He had difficulty hearing even when his ears were being directly shouted at. He was able to speak and communicate in writing and presented no aphasia. Otolaryngological examination found no abnormalities in the bilateral external auditory canal or tympanic membrane. On auditory brainstem response (ABR) testing on hospital day 16, poorly defined wave V was shown bilaterally indicating injury in the lower and central pons (Table 1 and Fig. 3). Antihypertensive therapy was continued and no exacerbations of hematoma or edema were observed on follow-up plain head CT. From around hospital day 20, bilateral hearing suddenly began to improve without intervention; however, a hearing test performed on hospital day 20, showed that bilateral hearing ability remained impaired at approximately 20 dB. The left tinnitus also improved and recovered to a level where it did not impede everyday conversation. He was transferred to a rehabilitation hospital on hospital day 26 due to residual dysarthria and right leg paralysis.

**Table 1:**
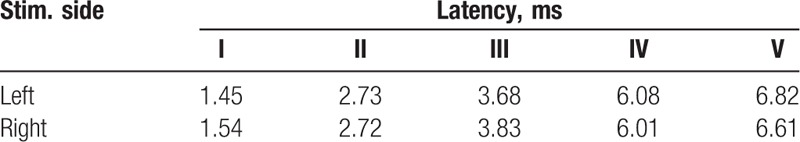
Auditory brainstem responses show poorly defined V waveforms.

## 3 Discussion

Post-stroke hearing loss can occur due to injury in the distribution of the anterior inferior cerebellar artery^[3]^; however, reports of this complication after pontine hemorrhage are rare.^[2,4–7]^ Hearing loss can be generally divided into 2 types: sensorineural and conductive. Sensorineural hearing loss accounts for about 90% of reported hearing loss and can be caused by damage to any of the structures of the auditory pathway: the cochlea, vestibulocochlear nerve, cochlear nucleus, temporal lobe, inferior colliculus, and auditory cortex. The auditory pathway begins with hair cells distributed in the organ of Corti in the inner ear. They pass information to spiral ganglia, which house the nerve cell bodies in the cochlea. The axons of these nerves form the cochlear nerve and pass through the inner ear together with the vestibular nerve, entering the brainstem laterally to the pons. Soon after entering the cranium, fibers from this vestibulocochlear nerve bifurcate, synapsing in the dorsal and ventral cochlear nuclei. From the cochlear nuclei, the fibers ascend bilaterally in the lateral lemnisci and enter the medial geniculate body. On the way, some fibers synapse in the trapezoid body or lateral lemniscus and, after exiting the trapezoid body, ascend in the central auditory pathway to communicate bilaterally with the inferior colliculus, thereby providing the auditory cortex with auditory information from both cochlear nuclei.^[8]^ Therefore, bilateral hearing loss in cases of cerebral injury or pontine hemorrhage such as the present patient is extremely rare. However, in cases of major hemorrhage that extends across the bilateral cochlear nuclei, hearing loss accompanied by disturbance of consciousness due to damage to the reticular formation may occur.^[9]^ In such cases, disturbance of consciousness is likely to prevent patients reporting the hearing loss.

To our knowledge, only 2 cases of acute onset bilateral hearing loss following pontine hemorrhage have been reported. In both cases, the hematoma was large and hearing loss developed some time after pontine hemorrhage onset. With regard to etiology, Kim et al proposed that the pontine hematoma injured the bilateral cochlear nuclei and trapezoid body,^[10]^ while Chung et al reported that the hematoma and surrounding edema compressed the bilateral cochlear nuclei.^[9]^ Compared to previous reports, the present case was characterized by the small hematoma volume and the absence of concurrent disturbance of consciousness at hemorrhage onset. Furthermore, in the present patient, bilateral hearing loss began on the day of hospital admission, which is earlier than in previous reports (Table 2). As hematoma volume was comparatively small in the present patient, it is possible that damage to the reticular formation was avoided, limiting marked damage to the auditory pathway.

**Table 2:**
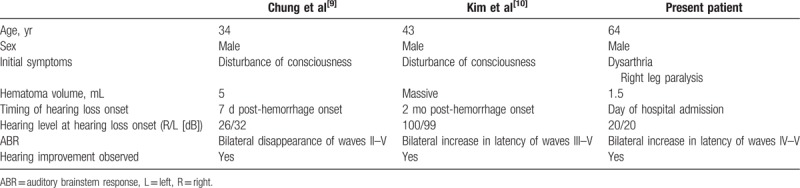
Comparison with previous reports.

The present study had several limitations. First, as the patient was transferred to another hospital immediately after spontaneous hearing recovery, ABR was not repeated. Second, at our hospital, we were unable to perform the otoacoustic emission test performed in previous reports.

In the present patient, the location of hematoma in the dorsomedial pons and the abnormalities in waves V on ABR indicate trapezoid body and lateral lemniscus injury. Furthermore, as symptoms improved with hematoma resolution, the injury was more likely to be in the form of indirect transient damage due to the compression by hematoma and surrounding edema rather than direct destructive damage to the auditory pathway. The predominant symptom in pontine hemorrhage is most commonly disturbance of consciousness caused by damage to the reticular formation and it is likely that any impaired hearing is overlooked in patients in this state. Therefore, care is required as latent communication disorder due to impaired hearing is possible regardless of the state of consciousness.

## Author contributions

**Conceptualization:** Masashi Hoshino.

**Data curation:** Hisanao Akiyama, Satoru Kashima, Kaima Soga, Takahiro Shimizu.

**Writing – original draft:** Masashi Hoshino.

**Writing – review and editing:** Hisanao Akiyama, Yasuhiro Hasegawa.

Masashi Hoshino orcid: 0000-0002-6798-0571.
